# The Role and Therapeutic Potential of miRNAs in Colorectal Liver Metastasis

**DOI:** 10.1038/s41598-019-52225-2

**Published:** 2019-11-01

**Authors:** Smiti S. Sahu, Shatovisha Dey, Sarah C. Nabinger, Guanglong Jiang, Alison Bates, Hiromi Tanaka, Yunlong Liu, Janaiah Kota

**Affiliations:** 1 Department of Medical and Molecular Genetics, Indiana University School of Medicine, Indianapolis, IN USA; 2 Department of BioHealth Informatics, Indiana University-Purdue University Indianapolis, Indianapolis, IN USA; 3 Department of Biochemistry and Molecular Biology, Indiana University School of Medicine, Indianapolis, IN USA; 4 The Melvin and Bren Simon Cancer Center, Indiana University School of Medicine, Indianapolis, IN USA

**Keywords:** Colorectal cancer, Cancer therapy

## Abstract

Colorectal cancer (CRC) is the fourth leading cause of cancer-related deaths worldwide. Liver metastasis is the major cause of CRC patient mortality, occurring in 60% patients with no effective therapies. Although studies have indicated the role of miRNAs in CRC, an in-depth miRNA expression analysis is essential to identify clinically relevant miRNAs and understand their potential in targeting liver metastasis. Here we analyzed miRNA expressions in 405 patient tumors from publicly available colorectal cancer genome sequencing project database. Our analyses showed miR-132, miR-378f, miR-605 and miR-1976 to be the most significantly downregulated miRNAs in primary and CRC liver metastatic tissues, and CRC cell lines. Observations in CRC cell lines indicated that ectopic expressions of miR-378f, -605 and -1976 suppress CRC cell proliferation, anchorage independent growth, metastatic potential, and enhance apoptosis. Consistently, CRC patients with higher miR-378f and miR-1976 levels exhibited better survival. Together, our data suggests an anti-tumorigenic role of these miRNAs in CRC and warrant future *in vivo* evaluation of the molecules for developing biomarkers or novel therapeutic strategies.

## Introduction

Colorectal cancer (CRC) is the fourth most common malignancy worldwide^1,2^. Liver metastasis occurs in >60% of CRC patients and is the major cause of their mortality^3–5^. Surgical resection and adjuvant therapies are the best available curative options for treating early stage liver metastases. However, most patients are diagnosed when the tumors have progressed to an unresectable stage, and these advanced tumors respond poorly to available therapies, highlighting the critical need to develop novel strategies for the treatment of colorectal liver metastases^2–5^.

MicroRNAs (miRNAs) are critical regulators of cellular homeostasis and gene expression^6^. The role of miRNAs in cancer pathogenesis has been well documented including CRC^7–9^. miRNAs are known targets of genomic lesions that frequently activate oncogenes and inactivate tumor suppressors in cancer cells by amplification, deletion and epigenetic silencing^10–12^. Additionally, miRNAs are known to modulate oncogenic and tumor suppressor signaling pathways^13–15^, and contribute to cancer cells proliferation, growth, invasion and metastasis^16,17^. Numerous functional studies have documented the pro^18–22^ and anti-tumorigenic activity^23,24^ of specific miRNAs in cancer cells and their potential use as novel anti-cancer agents^25–28^. Some miRNA-based drugs have already reached to phase 2 clinical trials^29^.

Here, we set out to conduct an in-depth study on a large cohort of CRC tumors to identify the clinically relevant miRNAs and assess their potential use to target liver metastasis. To this end, we analyzed 405 CRC patients’ miRNA expression data from the Cancer Genome Atlas (TCGA), and identified 58 miRNAs that are significantly downregulated in primary CRC tumors. Based on miRNA-gene target analysis and experimental validation in primary and metastatic CRC patient tumors, and CRC cancer cell lines, we show that restored expression of three miRNAs- miR-378f, miR-605 and miR-1976 reduces cancer cell proliferation, anchorage independent growth, migration, invasion, and induces cellular apoptosis, with profound impact on metastatic cells. This implicates their potential use as anti-cancer agents to target CRC metastasis. Because the selected miRNAs are associated either with colorectal liver metastasis, or with genomic loci that are commonly deleted in metastatic CRC, these miRNAs might have significant clinical relevance, or may be useful as biomarkers.

## Results

### Repression of miRNAs is a common feature of CRC

**Figure 1:**
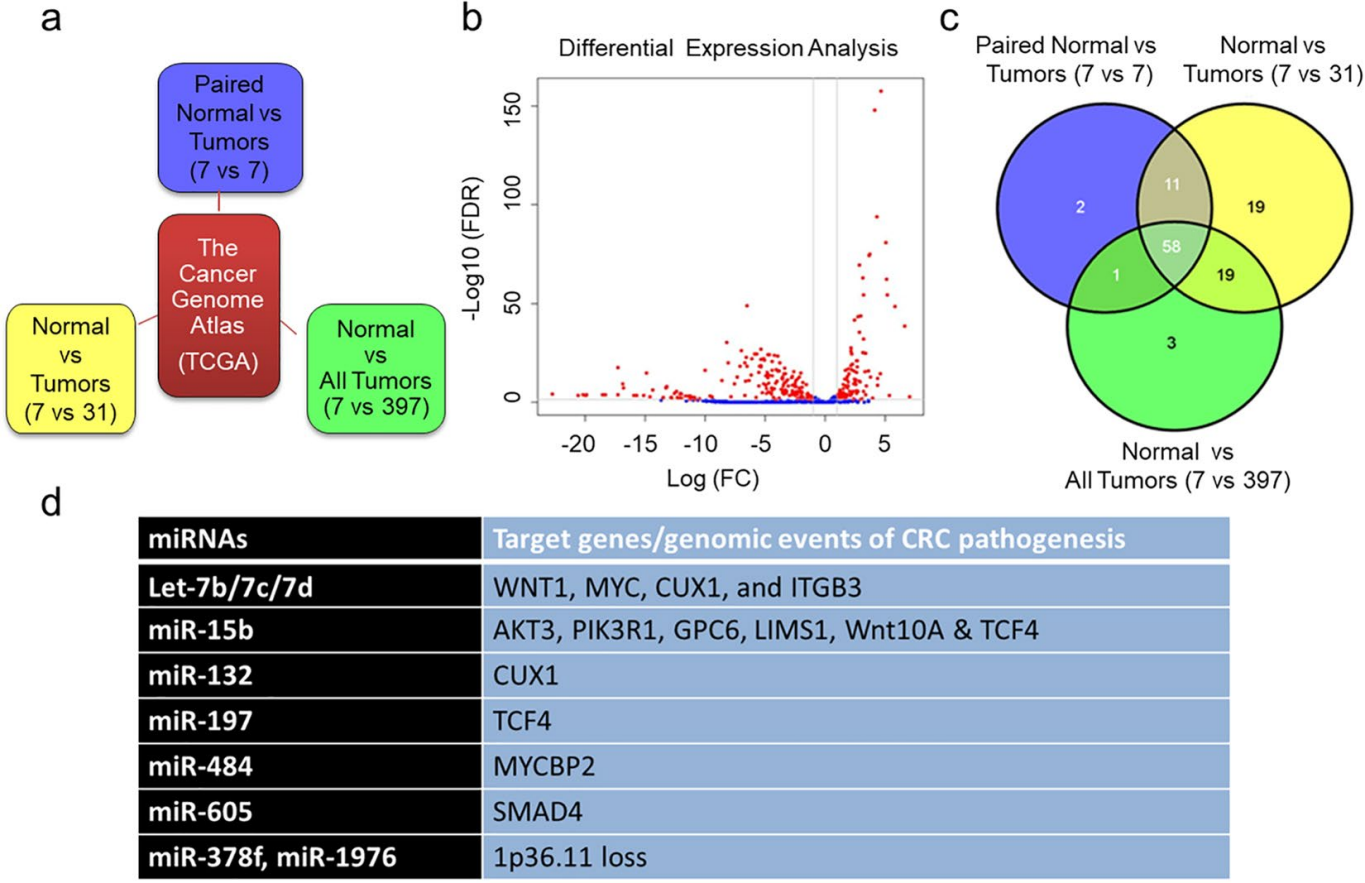
Repression of miRNAs is a common feature of Colorectal Carcinoma. Schematic of different datasets obtained from the cancer genome atlas (TCGA) for differential miRNA expression analysis between normal and primary Colorectal Carcinoma (CRC) tumors (**a**). Volcano plot for differential miRNA expression analysis of seven normal and 405 primary CRC tumors (**b**). Overlapping downregulated miRNAs in all three analyses with FDR 0.05 and LogFC value >1 (**c**). Significantly downregulated miRNAs in CRC tumors and their target genes or genomic events associated with CRC pathogenesis or liver metastases (**d**).

To identify the clinically relevant miRNAs that are differentially expressed in CRC tumors, we analyzed publicly available colorectal adenocarcinoma (COAD) sequencing project miRNA expression data from 405 CRC tumors covering 705 human miRNAs. We compared the miRNA expression patterns of seven normal colon tissues paired with CRC tumors, or with 31 tumors representing same analyte, or with 397 tumors (Fig. 1a). In all three comparisons (analyses), we calculated the Log2 Fold Changes (LogFC) and p-values for all 705 miRNAs to identify significantly misregulated miRNAs. To ascertain whether miRNAs expression patterns differentiate normal colon and CRC tumors, we performed principal component analysis (PCA) with all miRNAs detected, which separated the normal colorectal tissues and tumors into two clusters (Supplementary Fig. 1a–c). Comparisons of differential expression patterns in all three analyses show very consistent patterns in the expression change direction and significant levels (Supplementary Fig. 1d,e). As expected, global misregulation of miRNAs is a common feature of CRC tumors compared to normal colon tissue (Fig. 1b and Supplementary Fig. 2a,b). Consistent with previous studies^13,30^, although, some miRNAs are overexpressed, vast majority of the miRNAs are repressed in CRC tumors compared to normal colon (Supplementary Datasets 1–3). Since miRNA repression is a common feature of CRC tumors, we aimed to determine the functional role of downregulated miRNAs in the CRC pathogenesis; thus, our subsequent studies mainly focused on miRNAs that are downregulated in CRC tumors. Using this selection criterion, we identified 58 miRNAs common in all three analyses that were significantly downregulated (Log FC > 1.0 and FDR < 0.05) in CRC tumors compared to normal colon controls (Fig. 1c, Supplementary Dataset 4).

### miRNAs associate with molecular mechanisms or genomic alterations of colorectal liver metastasis

To further screen for the most clinically relevant miRNAs, and those that are associated with CRC liver metastasis, we analyzed the gene expression of the predicted targets of the 58 identified miRNAs from the TCGA COAD datasets and selected targets that are significantly downregulated with a p < 0.05 (Supplementary Dataset 5). Subsequently, we reviewed literature to check for any association of the predicated target genes with CRC progression/metastases. In parallel, we also analyzed genetic aberrations of downregulated miRNAs and their association with CRC metastases. Based on these analyses, we selected 10/58 miRNAs for further characterization, these include, Let-7 family members (Let-7b, -7c and -7d), miR-15b, -132, -197, -378f, -484, -605, and -1976 (Fig. 1d). Either the selected miRNAs regulate target genes involved in cellular mechanisms of cancer progression/metastases, or loss of their genomic regions is associated with CRC. Let-7 targets a number of genes that are involved in cell migration and invasion such as WNT1, MYC, CUX1^31^, and ITGB3^32^. WNT1 belongs to β-catenin signaling and MYC is a transcriptional target of this pathway; additionally, up-regulation of WNT1 is a common feature of CRC^8^. miR-15b, a bona fide tumor-suppressor miRNA, regulates a number of genes associated with CRC metastases such as AKT3, PIK3R1^8^, GPC6^33^, LIMS1^34^, and TCF4. CUX1 and TCF4 are predicted targets of miR-132 and miR-197 respectively and their upregulation is associated with CRC pathogenesis^35,36^. miR-484 targets MYCBP2, a member of the c-Myc oncogene family that plays a role in CRC pathogenesis^37^ and miR-605 targets SMAD4, a TGF-β1 family member whose upregulation is often associated with aggressive CRC^8^. In addition, miR-605 functions as tumor suppressor by positive regulation of p53^38^. Interestingly, miR-378f and miR-1976 are encoded in the same genomic locus, 1p36.11, loss of which is often associated with CRC metastasis^39^. Furthermore, miR-378f targets Wnt10A, a member of Wnt signaling and it is commonly upregulated in CRC^40^.

### Expression patterns of downregulated miRNAs in primary and colorectal liver metastasis tissue specimens and cell lines

**Figure 2:**
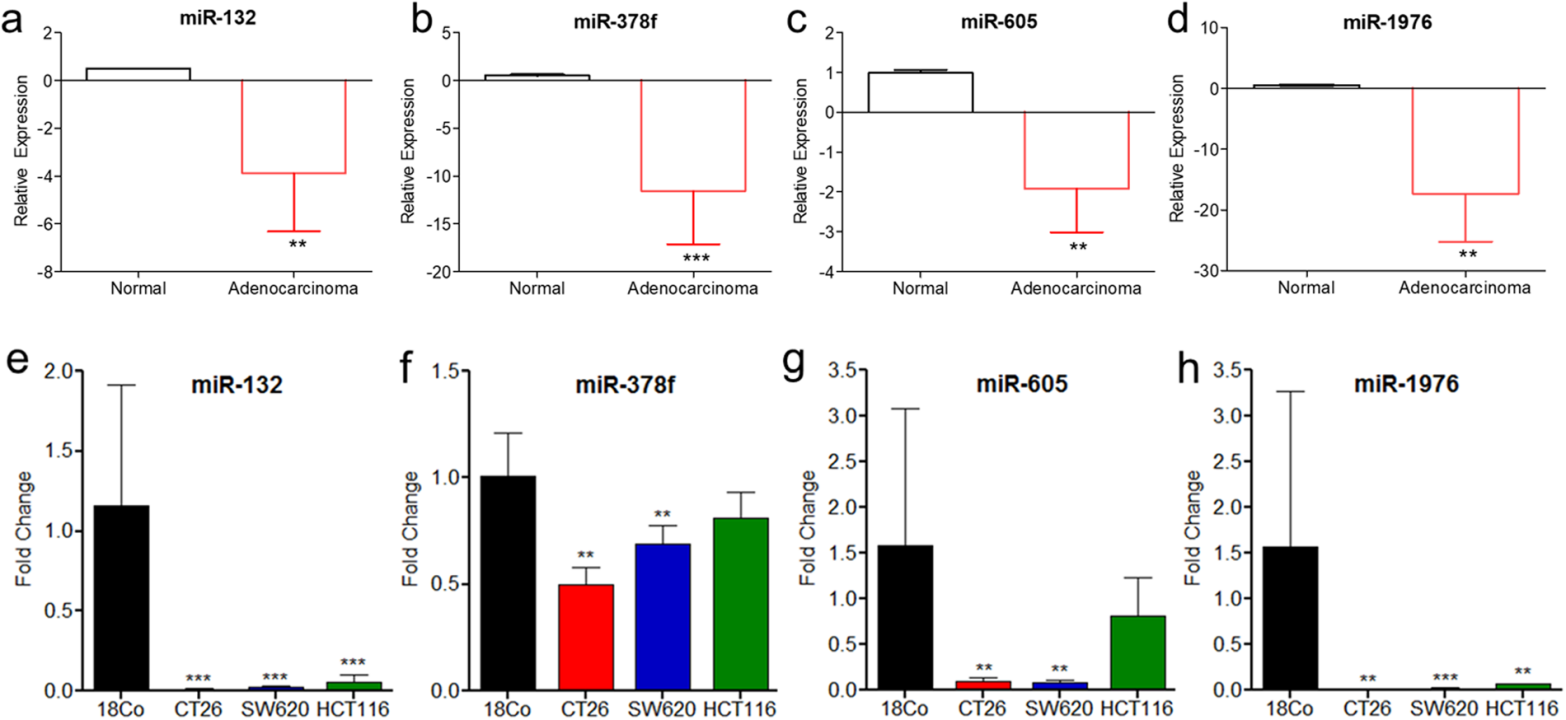
qPCR analysis of candidate miRNAs in CRC cell lines and patient tumors. Total RNA isolated from 15 paired primary colorectal tumors and normal colorectal tissue from CRC patients was analyzed by qPCR for miR-132 (**a**), miR-378f (**b**), miR-605 (**c**) and miR-1976 (**d**). Box plot graphs present relative expressions (fold changes) of primary colorectal adenocarcinoma (red) compared to normal adjacent tissue (black). N = 15; statistics computed by paired, student t-test; *p < 0.08, **p < 0.05, ***p < 0.01. Total RNA was isolated from normal colon (18Co), or CRC cell lines: CT26 (murine metastatic), SW620 (human metastatic), and HCT116 (human primary), and analyzed by qPCR for miR-132 (**e**), miR-378f (**f**), miR-605 (**g**), and miR-1976 (**h**) expression. Graphs represent average fold change + standard deviation; N = 3–4; statistics computed by student t-test; **p < 0.05, ***p < 0.009.

To further confirm the downregulation of 10 miRNAs in CRC, we determined the expression levels of 10 selected miRNAs using qPCR in 15 paired CRC primary tumors compared to adjacent normal colon controls (Supplementary Tables 1). Consistent with TCGA data, we observed significant repression of four selected miRNAs, miR-132, -378f, -605 and -1976 (Fig. 2a–d) and non-significant regulation of six miRNAs, Let-7b, -7c and -7d, -15b, -197, and -484 (Supplementary Fig. 2c–h). To further characterize the miRNAs that play a role in liver metastasis, we then validated the expression levels of the four selected miRNAs in seven paired primary and liver metastatic tumors compared to adjacent normal colon (Supplementary Table 2). All four selected miRNAs are downregulated >2-folds in ~29–57% of primary and metastatic tumors compared to normal adjacent controls of the same patient. For example, >2-fold downregulation of miR-132 and miR-605 was observed in 43% (3/7) patient primary colorectal tumor as well as liver metastasis tissues, miR-378f was downregulated in 57% (4/7) of both primary colorectal tumor and liver metastasis tissues, while miR-1976 was downregulated in 43% (3/7) primary colorectal tumor and 29% (2/7) liver metastasis tissues. To further confirm the loss of expression, we validated the expression levels of the four selected miRNAs in a primary colorectal cell line, HCT116 and in murine and human metastatic cell lines, CT26 and SW620 respectively compared to normal colon cell line, 18CO. In 18CO, although the expressions of miR-132, -605, -1976 exhibited some variation between experiments, the relative expressions in each individual experiment (n = 3) were consistent, and significantly differed from majority of the tested cell lines. Overall, our data indicated that the four miRNAs, miR-132, -605, -378f and -1976 were downregulated in both murine and human metastatic cell lines. Whereas, in primary colorectal cancer cell line, only miR-132 and -1976 were downregulated at statistically significant thresholds (Fig. 2e–h).

### Ectopic expression of repressed miRNA suppresses CRC cell proliferation and induces cell death

**Figure 3:**
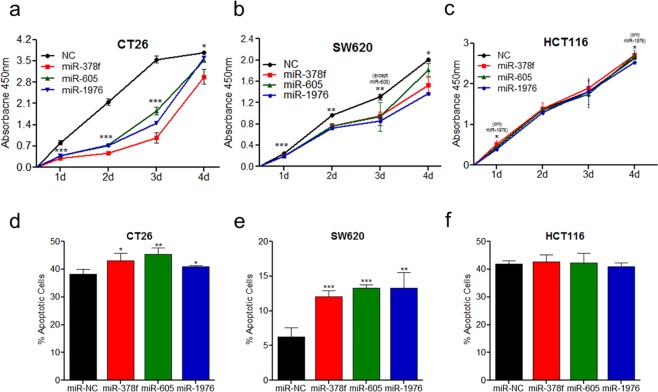
Restored expression of downregulated miRNAs inhibits cell proliferation and induces apoptosis in CRC cell lines. CT26 (**a**,**d**), SW620 (**b**,**e**), HCT116 (**c**,**f**) cell lines were transfected with miR-378f, miR-605, miR-1976, or negative control (NC) mimics and subjected to CCK-8 assays to measure proliferation for up to 4 days (**a**–**c**) or apoptosis assays at 72 hrs post-transfection (**d**–**f**). Apoptosis graphs represent total percentage of apoptotic cells that were stained positive for AnnexinV or 7-AAD. Data presented as average absorbance or % of apoptotic cells +/− standard deviation; N = 3; student t-test used to calculate p-values (p-value represents the largest p-value at each time point compared to the normal mimic control), *p < 0.05, **p < 0.009, ***p < 0.0009.

As consistent loss of miR-132, -378f, -605, and -1976 in primary and metastatic CRC tissues and cell lines was observed, we next assessed the anti-tumorigenic properties of these miRNAs in metastatic (CT26 and SW620) and primary (HCT116) CRC cell lines through miRNA gain-of-function studies. We transiently transfected CT26, SW620, and HCT116 cells with miR-132, -605, -378f, -1976 or control mimics and evaluated cell proliferation. Transfection efficiency of individual miRNAs was confirmed through qPCR in all three CRC cell lines (Supplementary Fig. 3a–c). In our studies, miR-132 transfected cells did not show difference in cell proliferation and death rate (Supplementary Fig. 3d–g) and thus we excluded miR-132 in subsequent functional analyses. Ectopic expression of miR-378f, -605, and -1976 in both mouse (CT26) and human metastatic (SW620) cell lines showed a significant decrease in cell proliferation (Fig. 3a,b). However, in primary CRC cell line, HCT116, only miR-1976 resulted in reduced cell proliferation at 1 and 4 days post-transfection, with no effect for miR-378f and miR-605 (Fig. 3c).

Subsequently, we validated cell death rates in CRC lines transfected with miR-378f, miR-605, and miR-1976. In both murine (Fig. 3d) and human metastatic (Fig. 3e) CRC cells, all three miRNAs, miR-378f, -605, and -1976 increased cell death rates. However, in primary CRC cell line HCT116, none of the miRNAs (miR-378f, -605 and -1976) induced cancer cell deaths (Fig. 3f).

### Ectopic expression of repressed miRNA inhibits cancer cell transformation

**Figure 4:**
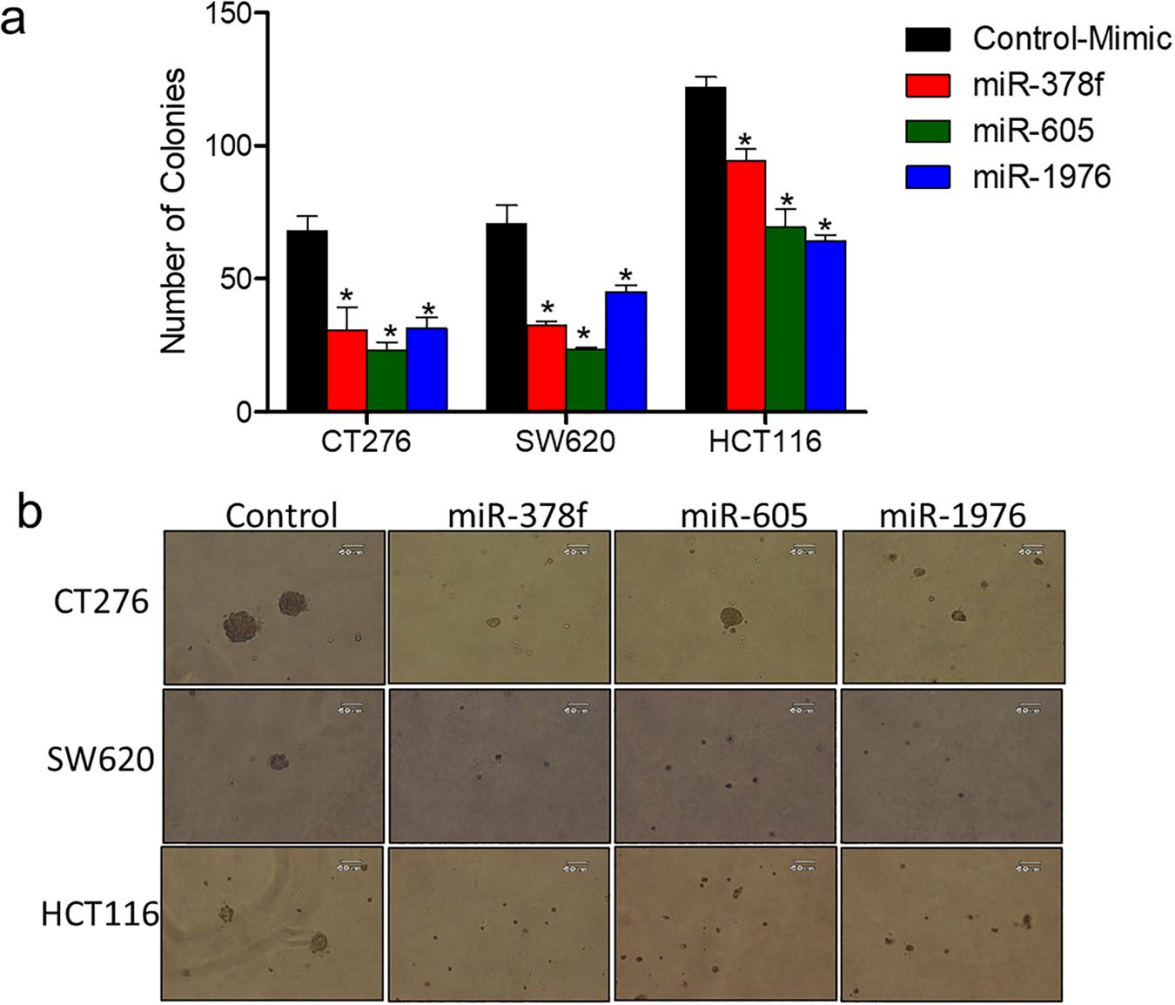
Restored expression of downregulated miRNAs inhibits anchorage independent growth of CRC cell lines. CRC cell lines (CT26, SW620, HCT116) were transfected with miR-378f, miR-605, miR-1976, or negative control (NC) mimics and plated into soft agar assays. Two weeks post plating, number of colonies were counted and presented as average number of colonies in 5 fields + standard deviation (**a**) with representative images of each below the graphs (**b**). Statistics generated using student t-test; N = 3, *p < 0.05.

As we observed reduced cell proliferation and increased cell death of metastatic CRC cell lines transfected with miR-605, -378f and -1976, we next assessed transformation potential of these miRNAs using anchorage-independent growth assays. Both metastatic (CT26 & SW620) and primary CRC (HCT116) (Fig. 4a,b) cell lines transfected with miR-378f, -605, and -1976 showed reduced transformation potential, with more robust effects on metastatic CRC cell lines compared to primary CRC cell line.

### miR-378f, miR-605, and miR-1976 expression inhibits migration and invasion of CRC

**Figure 5:**
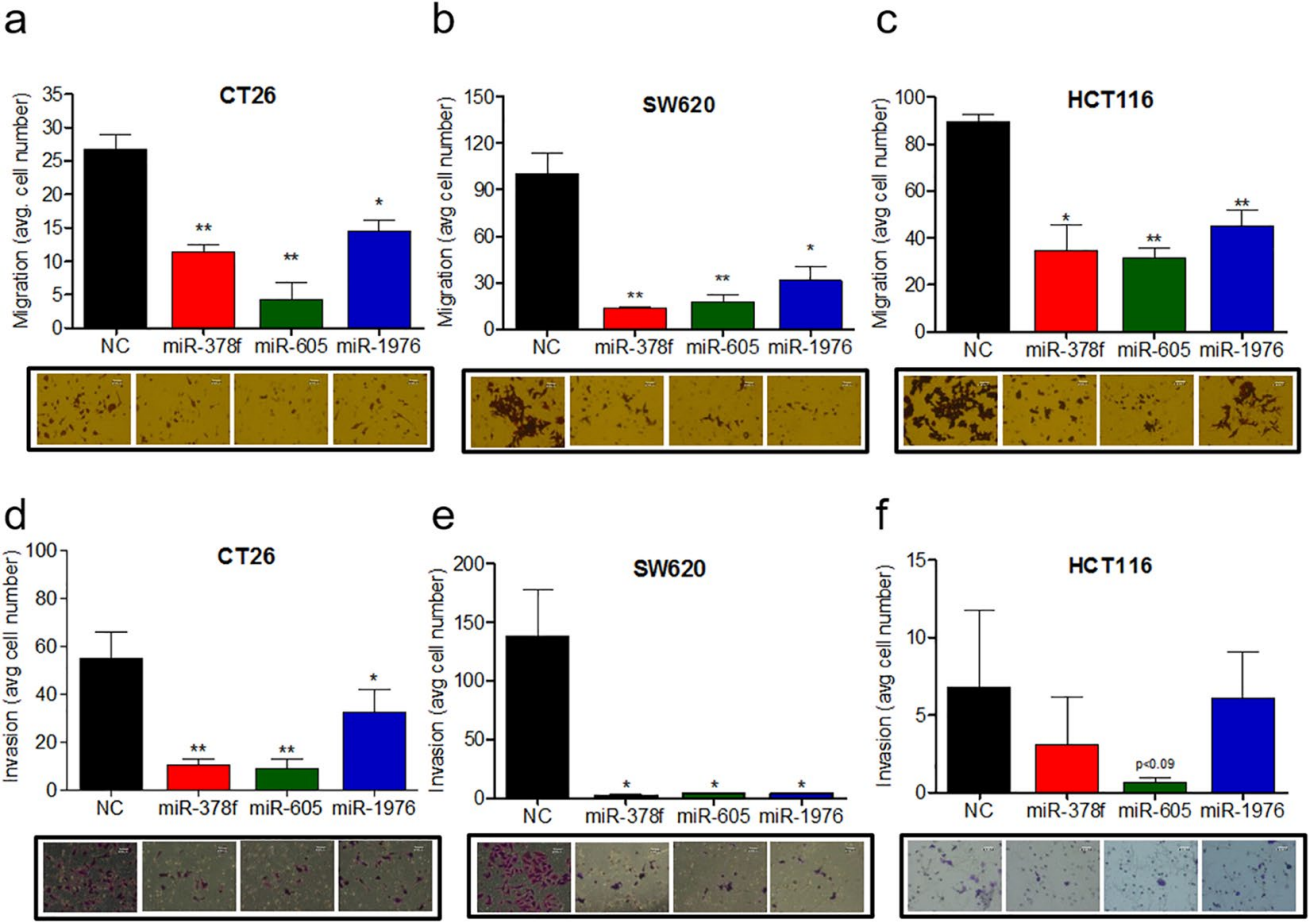
Restored expression of downregulated miRNAs reduce the ability of CRC cell lines to migrate and invade. CRC cell lines (CT26, SW620, HCT116) transfected with miR-378f, miR-605, miR-1976, or negative control (NC) mimics were plated into migration (**a**–**c**), and invasion (**d**–**f**) assays. Migration and invasion data presented as average number of cells in 5 fields + standard deviation with representative images of each below the graphs. Larger purple stained cells in the representative images signify migrated or invaded cells. Statistics generated using student t-test; N = 3, *p < 0.05, **p < 0.005.

Next, we evaluated the effects of ectopic expression of miR-378f, -605, and -1976 on migration and invasion potential of CT26, SW620 and HCT116 cells using transwell assays. Overexpression of all three down-regulated miRNAs (miR-378f, -605 and -1976) significantly inhibited cell migration of both metastatic CRC cell lines, CT26 and SW620 (Fig. 5a,b) and primary CRC cell line, HCT116 (Fig. 5c). Similarly, overexpression of miR-378f, -605, and -1976 suppressed CRC cells invasion into matrigel-coated transwell membranes in both metastatic cell lines (Fig. 5d,e). In primary CRC cell line, HCT116, which is less invasive in our experimental settings, none of the miRNAs was effective in reducing invasion potential (Fig. 5f).

### miR-378f and miR-1976 expression has better prognosis in CRC patients

**Figure 6:**
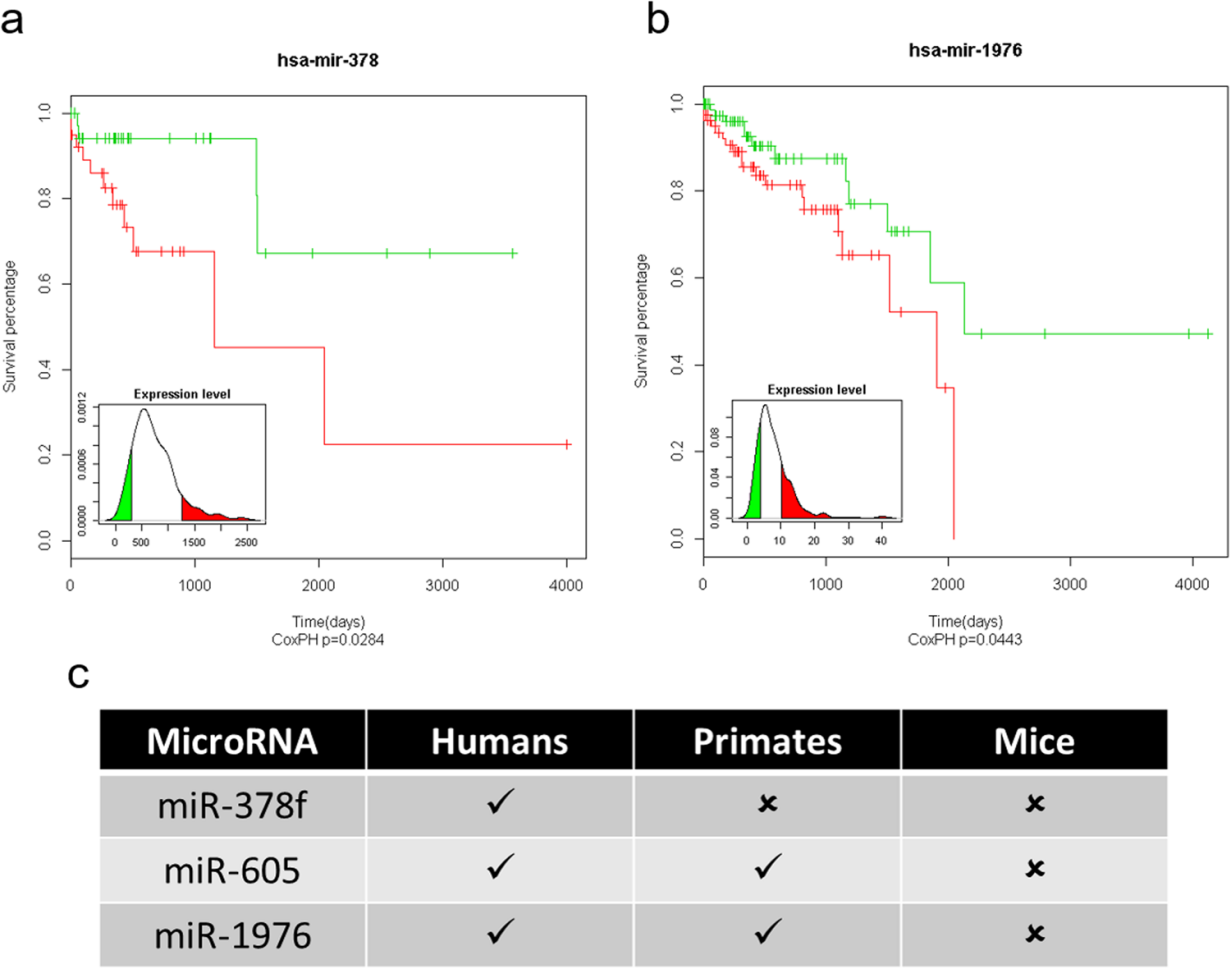
Higher miR-378f or miR-1976 expression levels correlate with increased overall survival of CRC patients. Clinical data from 406 patients was used to correlate miR-378f (**a**) and miR-1976 (**b**) expression levels with overall survival. For miR-378f, overall survival was correlated with the upper and lower 10% of miR-378f expression levels (P < 0.028). For the miR-1976 correlation analysis, the upper and lower 21% of miR-1976 expression levels were compared for overall survival (p < 0.044). Phylogenetic conservation of miR-378f, miR-605 and miR-1976 in humans, primates and mice (**c**).

To test the correlation of miR-378f, -605, and -1975 with clinical parameters, we performed correlation analysis using CRC patient data. Comparison of overall survival between patients expressing the top and bottom 10% of miR-378f expression revealed that patients with high miR-378f expression had better survival (p < 0.028, Fig. 6a). Similarly, patients expressing high miR-1976 (comparison of top and bottom 21%) had better survival (p < 0.044, Fig. 6b). Although, we observed ectopic expression of miR-605 to cause significant decrease in cell proliferation, migration and invasion, and increase in cell death, there was no correlation between miR-605 expression level and patient survival.

## Discussion

CRC is the fourth leading cause of cancer-related deaths worldwide^41^. Patients diagnosed early or with limited metastatic tumor burden have better prognosis with a variety of available treatment options such as surgery and/or in combination with chemo-radiotherapy^42^. However, patients with advanced metastatic disease have no effective treatment options^43^. Liver is the most common site of metastasis, which results in two-thirds of all CRC-related deaths^44^. Understanding the molecular mechanisms that regulate primary tumor progression and liver metastases will aid in developing novel therapeutic options for advanced disease. miRNAs regulate a wide variety of cellular mechanisms associated with cancer pathogenesis^45^. Restored expression of individual missing miRNAs reduced tumorigenesis in experimental models of various cancer types^28,46^. Based on comprehensive meta-analysis of publically available miRNAs expression data sets from 405 CRC patients, in the current study, we identified 10 miRNAs that are significantly downregulated in primary CRC tumors compared to normal colon tissues. Subsequent validation and functional studies of individual miRNAs in primary and metastatic CRC patient tissues and cell lines revealed three lead candidate miRNAs, miR-378f, -605 and -1976 that are significantly downregulated in both primary CRC and liver metastatic tumors. Restored expression of these three miRNAs reduced cell proliferation, metastatic phenotypes and induced cell death in both primary and metastatic CRC cell lines, with a profound impact on metastatic cells. Together, these data suggest the potential of the three miRNAs to serve as molecular signatures for predicting advanced CRC high-risk patients or in developing liver metastasis targeted miRNA therapeutic strategies. Nevertheless, these results need to be further corroborated functionally in clinical CRC specimens and their therapeutic use be validated in humanized murine models.

Interestingly all three colorectal liver metastases associated and functionally relevant miRNAs are less conserved and specific to non-human primates or humans (Fig. 6c). Specifically, miR-378f is the most poorly conserved miRNA among the three and has been annotated only in the human genome. Deletion of the chromosomal region encoding miR-378f, 1p36.11, is often associated with CRC metastasis^39^. Consistently, large-scale miRNA profiling studies and CRC tumors expressing oncogenic Kras mutations repress miR-378f and other family members^47–49^. Particularly, circulating miR-378 is downregulated in stage II CRC tumors^50^ and associated with poor prognosis^51,52^. Further, functional studies document that miR-378 regulates of variety of genes associated with cell proliferation, invasion, migration and EMT phenotypes^53^. These reports implicate that miR-378 could potentially be used as a biomarker or therapeutic agent for CRC.

miR-1976 is also poorly conserved in humans and non-human primates, and limited reports are available on the role of this miRNAs in cellular processes. In particular, there are mixed reports about the role of miR-1976 in carcinogenesis. In small lung cancer, miR-1976 functions as a tumor suppressor^54^. In contrast, upregulation of miR-1976 correlates with poor survival in endometrial carcinoma patients^55^. Furthermore, miR-1976 has also been reported to negatively regulate tumor suppressor p53^56^. Nevertheless, our functional studies document a tumor suppressor function of miR-1976 in CRC. Further investigation will aid to dissect the mechanistic role of this miRNA in CRC pathogenesis.

miR-605 is a positive regulator of the p53 network. It is transcriptionally activated by p53 and is known to repress MDM2, a negative regulator of the p53 pathway. A large of body of data examining WT p53-expressing cancer cells, imply that miR-605 interrupts p53:MDM2 interactions and creates a positive feedback loop for rapid accumulation of p53^57^. We found significant downregulation of miR-605 in CRC tumors and its restored expression induced robust cell death in CRC cell lines. It is possible that miR-605 induces apoptosis by repression of MDM2 and enhances p53 function in CRC cells. In addition, we also found that miR-605 reduces invasive phenotypes of CRC cells. Our functional data implicates that a combination of its positive feedback regulation of the p53 axis and negative regulation of pro-metastatic genes likely confer the tumor suppressive function of miR-605 in CRC. However, these axes need to be validated *in vitro* and *in vivo* to exploit miR-605 for therapeutic applications in CRC.

In conclusion, our comprehensive miRNA expression analysis in a large cohort of CRC samples followed by functional studies implicates that three candidate miRNAs- miR-378f, -605 and -1976 potentially function as tumor suppressors and reduce invasive potential of CRC. Our results also implicate that validation of three miRNA signatures in a large set of CRC samples may help affirm their use as biomarkers for CRC patients who are at risk for metastatic disease. However, to fully decipher the functional relevance of these miRNAs in CRC, it would be essential to develop future *in vivo* functional studies, and evaluate tumor progression and liver metastasis associated with each candidate miRNA. Our current assessment of these miRNAs, complemented with verification in pre-clinical models, will potentially form the basis for developing novel miRNA based therapeutic strategies to reduce metastatic disease burden in CRC.

## Materials and Methods

### Public datasets

miRNA, mRNA sequencing, and clinical data for COAD patients were downloaded from TCGA data portal. miRNA sequencing of 705 miRNAs was available for 405 samples. Among 405 samples, 397 are primary tumors, seven are normal tissues and one is recurrent tumor. Of 397 primary tumors, tumor and matched normal tissue data sets were available for seven samples. At the time of data download, mRNA-seq was available for 211 samples, among which 193 samples overlapped with miRNA-seq dataset. The level 3 processed read count data for miRNA and mRNA expressions were downloaded. Clinical data for 439 samples were available and 303 samples overlapped with miRNA-seq data.

### TCGA data analyses

#### miRNA Differential Expression

Differential expression analyses were conducted with edgeR package in statistical environment R between normal and primary tumor samples. The expression abundance of miRNAs were compared between a) seven paired primary tumors and normal tissues; b) seven normal tissues and 31 primary solid tumors, which came from the same analyte of RNA samples; c) seven normal tissues and all 394 available primary solid tumor samples. The generalized linear model was adapted for the test of paired samples, and the exact test was used for the other two tests. Total 705 miRNAs identified by HiSeq were used in the differential expression analysis. The Benjamini-Hochberg Procedure was used to correct for the multiple comparisons and FDR <0.05 was considered as statistically significant.

#### miRNA target prediction

The links between miRNA and its target mRNA were evaluated in two ways. First, the correlations between miRNA and mRNA expression were tested with TCGA sequencing datasets as stated above. The normalized expression data from TCGA for both miRNA-seq and mRNA-seq on 193 common samples were used, and the negative-binomial regression (MASS package in R) and the spearman’s rank correlation were fitted to model the correlation between miRNA and its target genes. The Benjamini-Hochberg Procedure was used to correct for the multiple comparison and FDR <0.05 was considered as statistically significant. Only negative correlations were considered for further analyses. Second, only the genes that are predicted targets of the identified miRNAs, determined using TargetScan, were included for further assessments. All the statistical analyses were conducted in R environment, and snowfall package (https://CRAN.R-project.org/package=snowfall) was used for parallel computing.

#### Prognosis of miRNA

To evaluate the prognosis power of the miRNAs in COAD patients, Kaplan Meier curve was used to analyze the difference in overall survival probabilities in populations with high and low expression levels of miRNAs. Cox Proportional-Hazards Model was used to evaluate the statistical differences between two groups and p-value < 0.05 was considered statistically significant. The statistical analyses were conducted using Rv3.0.

#### Clinical samples

Paired formalin fixed paraffin embedded (FFPE) tissue samples from patients with CRC with and without liver metastasis were used to isolate total RNA and quantify miRNA levels. All experimental procedures were performed according to National Institute of Health (NIH) guidelines under protocols approved by the Indiana University-Purdue University Indianapolis (IUPUI) Institutional Review Board (IRB) (IUPUI IRB# 1303011057), which procures tissue samples. Informed consent was obtained from human subjects in compliance with IUPUI IRB policies. IUPUI IRB is accredited by the Association for the Accreditation of Human Research Protection Programs (AAHRPP). Quality of FFPE or fresh CRC tissue biopsies were analyzed by a certified pathologist with hematoxylin and eosin staining. Control samples, biopsied from patients with conditions unrelated to CRC or from an unaffected area of the colon or rectum from patients with CRC, were confirmed via histopathological analysis. Corresponding clinical data for each sample is provided in the Supplementary Tables 1 and 2.

#### RNA extractions

Total RNA was extracted from FFPE samples (2, 10uM thick sections) using the MagMax^TM^ FFPE total nucleic acid isolation kit (Life Technologies) or from frozen tissues using Rino BulletBlender beads (MidSci), or from cells using Trizol (Life Technologies) following manufacture’s protocols. RNA quantity was determined using a Nanodrop 2000.

#### qPCR analysis

Mature miRNA levels were measured by TaqMan miRNA Assays specific for let-7b (#478576_mir), -7c (#478577_mir), -7d (#477848_mir), miR-15b (#477929_mir), -132 (#000457), -197 (#477959_mir), -378f (#462794_mat), -484 (#478308_mir), -605 (#478174_mir), and -1976 (#478365_mir) purchased from Thermo Fisher Scientific using U6 snRNA (#Hs00984809_m1) as the reference to normalize the relative amount of miRNA in each sample. Samples were run in triplicates using an ABI 7500 qPCR machine and threshold of 0.2 was used for the analysis. Fold change was determined using the ΔΔCT method.

#### Cell lines

Colorectal carcinoma cell lines, CT26, SW620 and HCT116 were obtained from ATCC and grown in RPMI (CT26), L-15 (SW620) or McCoy’s (HCT116) supplemented with 10% fetal bovine serum and 1% penicillin/streptomycin.

#### Mimic transfection

Exponentially growing CT26, SW620, or HCT116 were seeded in six well plates at 2 × 10^5^ cells/well and transfected with 50–100 nM miRNA mimics using DharmaFECT®1 per manufacturer protocol. 24–48 hours post-transfections, cells were serum starved overnight and then plated into endpoint assays. Mimics were obtained from Life Technologies: control (CN-001000-01), miR-132 (C-301052), miR-378f (C-301994), miR-605 (C-300930), and miR-1976 (C-301473).

#### Proliferation, apoptosis, migration and invasion

CRC cell lines were transfected and serum starved as previously described^46^. 1–5 × 10^3^ cells were plated in 96-well plates in triplicate and grown at 37 °C. Proliferation was measured 24–120 hours after plating the assays by adding 10 ul Cell Counting Kit-8 (Dojindo #CK04) reagent and absorbance measured 1–4 hours later (450 nM). Apoptosis was quantified by flow cytometric analysis of the percentage of cells that bound Annexin V and 7-AAD (BD Biosciences kit) following manufacturer’s instructions. Migration and invasion assays were performed as previously described^58^ and quantified by counting five fields/membrane.

### Soft agar colony formation assay

The soft agar assay was performed in six-well plates containing two layers of Sea Plague Agar (Invitrogen). The bottom layer consisted of 0.8% agar in 1 ml of McCoy’s 5A Medium with 10% FBS. Cancer cells (1 × 10^3^/well) were placed in the top layer containing 0.7% agar in the same medium as the bottom. Cells were cultured for 14 days and colonies were photographed under a microscope.

### Statistical analysis

Student’s t-test was used for statistical analysis. Error bars are represented as standard deviation.

## Supplementary information

Supplementary Tables and Figures

Dataset 1

Dataset 2

Dataset 3

Dataset 4

Dataset 5
