# Supplementary material for: The Role and Therapeutic Potential of miRNAs in Colorectal Liver Metastasis
Source: Sci Rep. 2019 Nov 1;9:15803. doi: 10.1038/s41598-019-52225-2 (PMC6825151; doi:10.1038/s41598-019-52225-2)
Supplement: Supplementary file 1 — Supplementary Tables and Figures [file 41598_2019_52225_MOESM1_ESM.pdf]

The Role and Therapeutic Potential of miRNAs in Colorectal Liver Metastasis

Smiti S. Sahu[1], Shatovisha Dey[1], Sarah C. Nabinger[1], Guanglong Jiang[1,2], Alison Bates[3], Hiromi Tanaka[1], Yunlong Liu[1], and Janaiah Kota[1,4*]

Supplementary Table 1. CRC patient data (N=15) for specimens with primary tumor and normal adjacent tissues.

	**Colorectal Cancer(CRC)**
**Colon/Rectum**	
Normal Adjacent	15
Tumor	15
**Sex**	
Male	9
Female	6
**Age at procedure**	
Range	47-89
**Race**	
Non-Hispanic, White	15
**Degree of differentiation**	
Well	1
Moderate	9
Poor	3
**TNM Staging**	
Depth of tumor	
T0	0
T1	2
T2	0
T3	10
T4	3
Necrosis/ Lymph node status	
N0	8
N1	3
N2	4
N3	
Distant Metastases	
M0	0
MX	15
M1	0

Supplementary Table 2. CRC patient data (N=7) for specimens with primary tumor, normal adjacent tissues and liver metastasis.

Patient	Gender	Age	Ethinicity/Race	Specific site	Other body site	Depth of tumor	Necrosis/Lymph node status	Distant Metastases
P1	M	57	White	Colon	Sigmoid colon,Liver	T3	N2a	M1
P2	F	66	White	Rectum	Sigmoid colon,Liver	T3	N2	MX
P3	F	59	White	Colon	Colon, Liver	T3	N2a	M1
P4	M	74	White	Colon	Rectum, Liver	T2	N0	M1
P5	M	36	White	Rectum	Rectum, Liver	T3	N0	MX
P6	F	69	White	Colon	Colon, Liver	invasive metastases		
P7	F	54		Colon	Colon, Liver	not available		

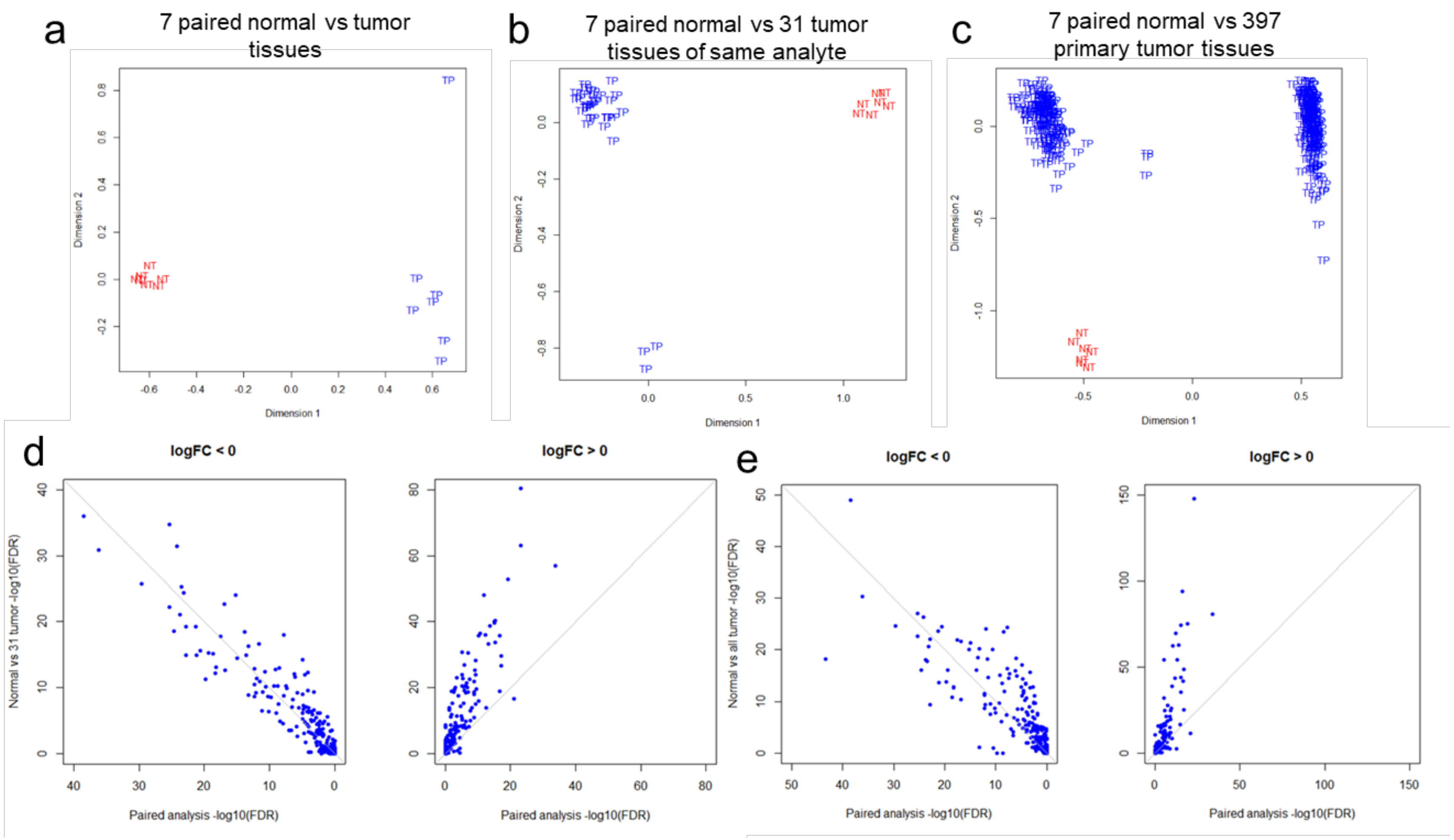

Supplementary Figure 1: Comparative differential expression analyses. Scatter plots for principal component analysis (PCA) of miRNA expressions from 7 paired primary colorectal carcinoma tumors and normal colorectal tissues depicting linear combinations of the tumors and normal tissues, that separate out different clusters **(a-c)**. Scatter plots with –logFDR values for differential expression analyses showing consistence between two different studies comparing seven paired normal tissues vs. tumors **(d)**, and 7 normal tissues vs. 397 primary tumors **(e)**.

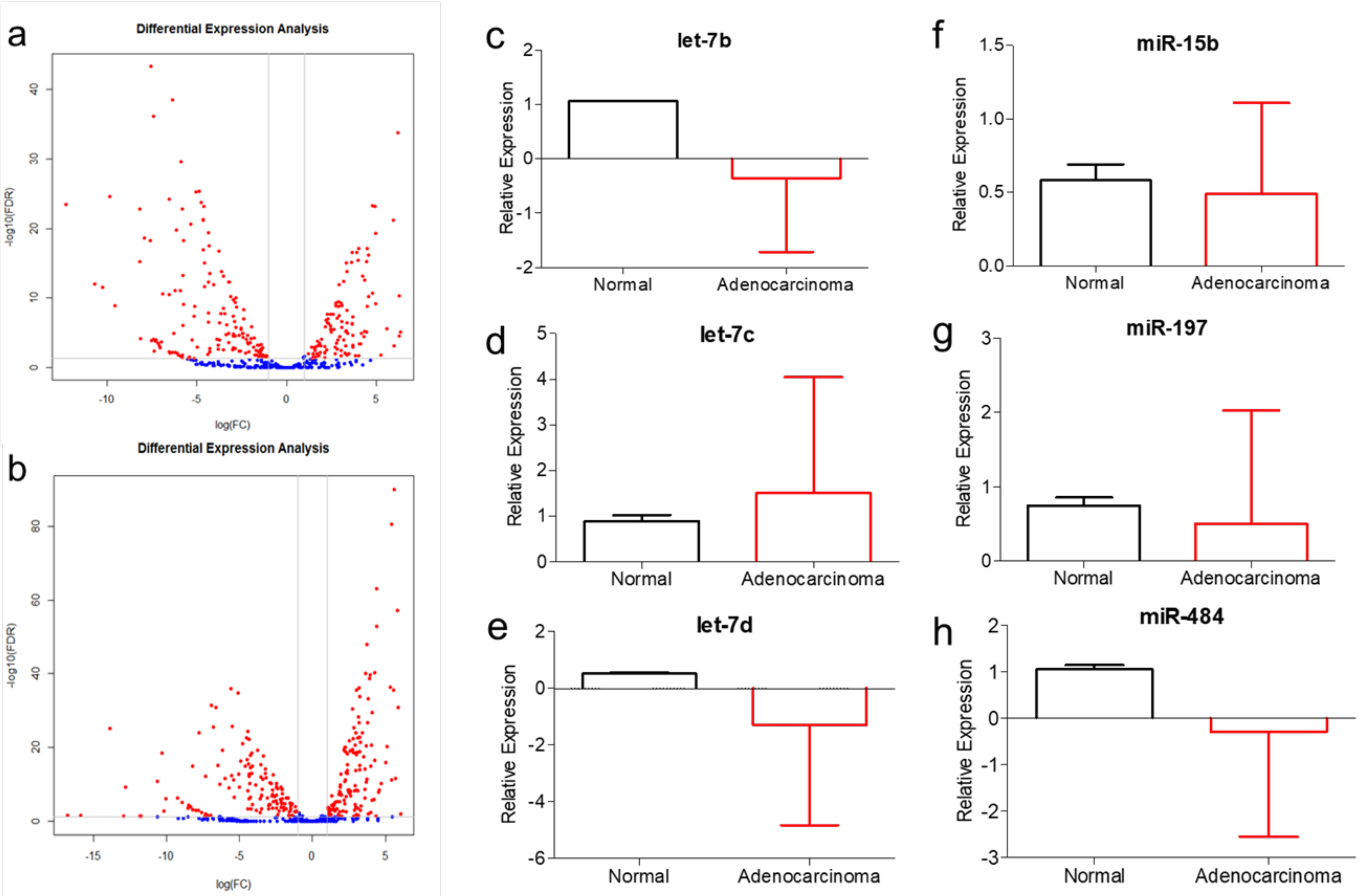

Supplementary Figure 2: Differential and relative expressions of miRNAs in normal vs. paired CRC tumors. Volcano plots for differential miRNA expression analysis of 7 Paired normal tissues vs. tumors (**a**), and 7 normal tissues vs. 31 tumors of same analyte (**b**). Relative expressions of miRNAs from total RNAs isolated from colorectal tumors and normal adjacent tissues obtained from CRC patients analyzed by qPCR for let-7b (**c**), let-7c (**d**), let-7d (**e**), miR-15b (**f**), miR-197 (**g**), miR-484 (H). Box plot graphs present relative expressions (fold change) of primary adenocarcinoma (red) compared to normal adjacent tissue (black). N=15; statistics of fold change was computed by paired, student t-test (all p-values were non-significant (between 0.1 and 0.5)).

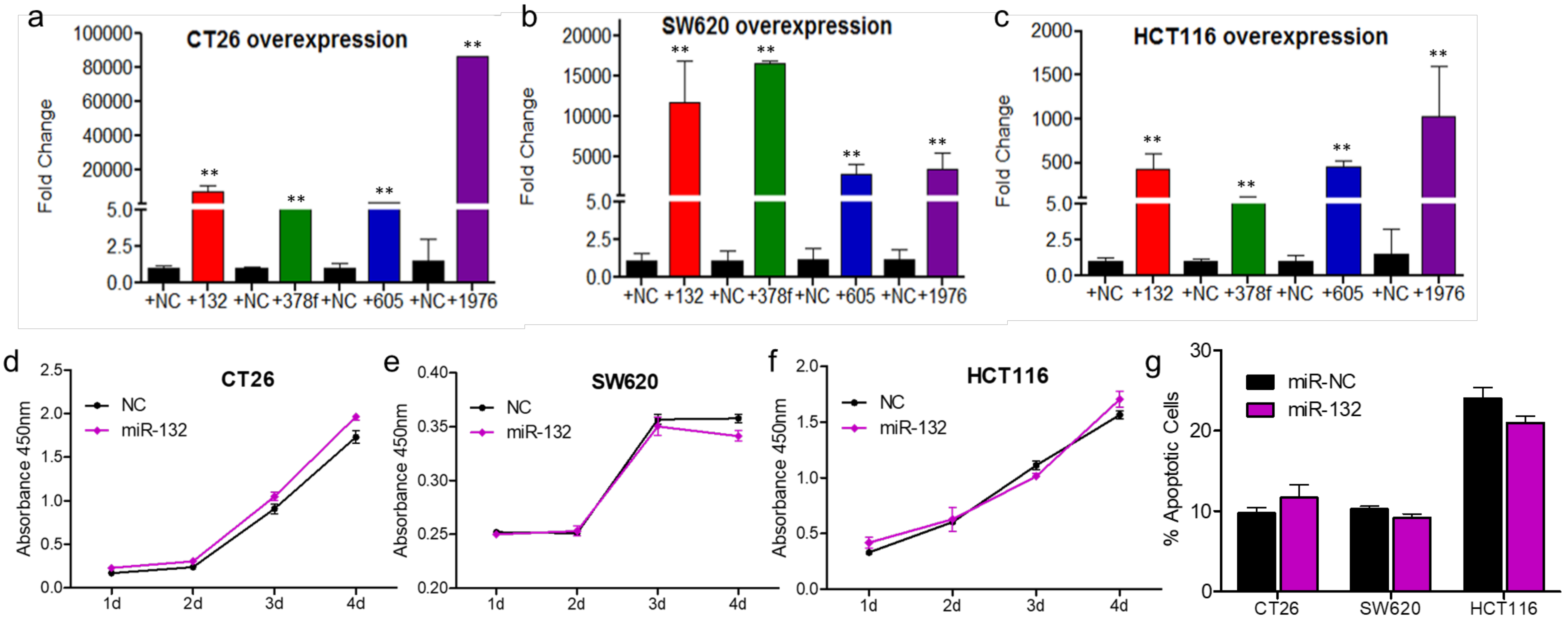

Supplementary Figure 3: Functional validation of identified miRNAs in CRC cell lines. CT26 (**a**), SW620 (**b**), HCT116 (**c**) cells transfected were transfected with 25nM control, miR-132,-378f, -605, and 1976 mimics, total RNA was extracted and expression levels were measured by qPCR using U6 snRNA as internal control. Cell proliferation rate for ectopic expression of miR-132 in CT26 (**d**), SW620 (**e**), and HCT1116 (**f**) cells. Cellular apoptosis rate for ectopic expression of miR-132 in the three CRC cell lines (**g**). Data is presented as mean ± SEM; n=3, Statistics calculated using student t-test, **p<0.01.
